# Metabolic Imaging of Head and Neck Cancer Organoids

**DOI:** 10.1371/journal.pone.0170415

**Published:** 2017-01-18

**Authors:** Amy T. Shah, Tiffany M. Heaster, Melissa C. Skala

**Affiliations:** 1 Department of Biomedical Engineering, Vanderbilt University, Nashville, TN, United States of America; 2 Department of Biomedical Engineering, University of Wisconsin, Madison, WI, United States of America; 3 Morgridge Institute for Research, Madison, WI, United States of America; University of South Alabama Mitchell Cancer Institute, UNITED STATES

## Abstract

Head and neck cancer patients suffer from toxicities, morbidities, and mortalities, and these ailments could be minimized through improved therapies. Drug discovery is a long, expensive, and complex process, so optimized assays can improve the success rate of drug candidates. This study applies optical imaging of cell metabolism to three-dimensional *in vitro* cultures of head and neck cancer grown from primary tumor tissue (organoids). This technique is advantageous because it measures cell metabolism using intrinsic fluorescence from NAD(P)H and FAD on a single cell level for a three-dimensional *in vitro* model. Head and neck cancer organoids are characterized alone and after treatment with standard therapies, including an antibody therapy, a chemotherapy, and combination therapy. Additionally, organoid cellular heterogeneity is analyzed quantitatively and qualitatively. Gold standard measures of treatment response, including cell proliferation, cell death, and *in vivo* tumor volume, validate therapeutic efficacy for each treatment group in a parallel study. Results indicate that optical metabolic imaging is sensitive to therapeutic response in organoids after 1 day of treatment (p<0.05) and resolves cell subpopulations with distinct metabolic phenotypes. Ultimately, this platform could provide a sensitive high-throughput assay to streamline the drug discovery process for head and neck cancer.

## Introduction

Head and neck cancer describes malignant tumors in the mouth, nose, and throat. Current treatments include chemotherapy, surgery, radiation therapy, and targeted therapy. Despite advancements in therapies, the 5-year survival rate for head and neck cancer is between 40–50% [1]. Additionally, chemotherapy, surgery, and radiation therapy introduce major toxicities, including damage to tissue and organs in anatomical sites that are critical for breathing, eating, and talking [2]. Therefore, organ preservation is an important consideration to maintain normal function. Targeted treatments for head and neck cancer focus on inhibition of the epidermal growth factor receptor (EGFR), particularly with the anti-EGFR antibody cetuximab [3]. However, there is a lack of targeted therapies beyond EGFR inhibitors. Additionally, tumor heterogeneity can allow a minority population of cells to drive treatment resistance and tumor recurrence [4]. Optimized therapies could provide better treatment efficacy and reduced toxicities, leading to improved quality of life and longer survival, but drug development takes at least 10 years and more than $1 billion [5][6]. Therefore, more accurate rapid drug screens to identify the most promising drug candidates and combination treatments would increase the success rate during drug development and facilitate the commercialization of optimized drugs and combinations.

*In vitro* three-dimensional cultures grown from primary tumor tissue (organoids) are attractive for a high-throughput drug screen that enables testing of multiple drugs and drug combinations. Cellular level measurements can identify cell subpopulations that exhibit different sensitivities to treatments, and organoids combined with high-resolution imaging of cell metabolism provides a promising platform. Organoids are physiologically relevant because they grow in a three-dimensional organization, are generated from tumor tissue, and can therefore capture distinct behaviors of individual tumors [7]. Additionally, multiphoton microscopy of cell metabolism has been shown to resolve therapeutic response in cancer [8][9], and the spatial scales of this imaging technique allow the full volume of the organoid to be imaged on a single-cell level. Autofluorescence measurements of the metabolic cofactors NAD(P)H and FAD characterize cell metabolism using their fluorescence intensities and lifetimes [10][11]. NAD(P)H and FAD autofluorescence can be measured by optimizing the excitation and emission wavelengths for these molecules. In cancer cells, the primary fluorescence signal in these channels would result from NADH and FAD, respectively. However, other cell types could include other molecules that interfere with these channels. In particular, keratin, collagen, and vitamins A, K, and D could be present in the NAD(P)H channel, and lipofuscin could be present in the FAD channel [12]. Cyanide perturbations have verified that the dominant signal in the NAD(P)H channel is NADH, and the dominant signal in the FAD channel is FAD in tumor cells [9][8]. This perturbation is known to increase NADH levels and decrease FAD levels [13], and our measurements confirmed these trends in head and neck cancer with the imaging parameters used in the current study [9]. This is expected because of the spectral properties, quantum yield, and concentration of NADH and FAD relative to these other possible contributors [14][15]. The fluorescence intensity measures relative amounts of each cofactor and the optical redox ratio, defined as the fluorescence intensity of NAD(P)H divided by that of FAD, reflects global cell metabolism. The fluorescence lifetime measures the amount of time a molecule is in the excited state, reflects protein-binding, and is sensitive to cellular signaling pathways that use NAD(P)H and FAD. Metabolic imaging based on cellular autofluorescence provides early, sensitive measurements of anti-cancer treatment response [8].

Organoids have been established and characterized for some types of cancers, including breast cancer and pancreatic cancer [16][17]. Different anatomical sites exhibit different cell types, cell structures, media and growth factor requirements, matrix stiffness requirements, and tissue digestion protocols, so characterization of each tumor type is necessary. Previous studies have focused on culturing spheroids from head and neck cancer cell lines [18], organoids from human salivary glands [19], and tumor pieces from head and neck cancer patients [20]. Therefore, characterization and analysis of head and neck cancer organoids grown from primary tumor tissue would be a new and beneficial contribution.

The lack of targeted treatments for head and neck cancer justify the need for a high-throughput drug screen. This study applies metabolic microscopy to non-invasively characterize head and neck cancer organoids alone and in response to drug treatments. Overall, this technique could be applied to streamline drug discovery and enable the development of optimized therapies with high efficacy and low toxicity.

## Materials and Methods

### Tissue Culture and Tumor Inoculation

FaDu cells were acquired from the American Type Culture Collection (ATCC HTB-43). FaDu cells were grown in Dulbecco’s Modified Eagle Medium (DMEM) plus 10% fetal bovine serum (FBS) and 0.4 μg/mL hydrocortisone. Animal work was approved by the Vanderbilt University Institutional Animal Care and Use Committee (IACUC). Mice were in sterile housing and were checked daily to ensure well-being and sufficient food and water. Subcutaneous flank tumors were inoculated in nude mice with 10^7^ FaDu cells. Tumors were grown for 1–2 weeks until reaching a volume of ~100mm^3^. For *in vivo* imaging the mouse was anesthetized using 2% isoflurane, the tumor was exposed, and the mouse was placed on the microscope. Organoid media consisted of DMEM plus 10% FBS, 0.4 μg/mL hydrocortisone, 1% penicillin: streptomycin, insulin-transferrin-selenium at a 1X concentration, 10ng/mL epidermal growth factor, and B27 at a 1X concentration. Treatment media for organoids included cetuximab (20nM) [21], cisplatin (33μM) [22], or their combination.

### Organoid Generation

Mice were anesthetized using 2% isoflurane and tumors were excised and immediately placed in chilled culture media. Tumors were washed 3 times with sterile phosphate buffered saline (PBS), transferred to 35mm petri dishes with 0.5mL culture media, and mechanically digested with scissors. Digestion into a cellular suspension was confirmed with brightfield microscopy. The cell suspension was mixed with matrigel at a volume ratio of 1 part cell suspension to 2 parts matrigel, and 100μL was plated on each 35mm glass-bottomed imaging dish (MatTek). The gels solidified at room temperature for 30 minutes and then at 37C for 1 hour. Then 2mL organoid media was added to each dish and organoids were grown at 37C.

### Tumor Growth Curves and Immunohistochemistry

Mice with FaDu tumors were treated 3 times a week for 2 weeks with cetuximab (33mg/kg) [23][24], cisplatin (6mg/kg) [25], or their combination via intraperitoneal injection (6 tumors per group). Tumor volumes were measured once a day using calipers, and tumor volumes were calculated by (l*w^2^)/2, where l is the tumor length and w is the tumor width. Tumor volumes were normalized to the size on day 1. On day 11 for the combination treated mice or day 13 for the single agent treated mice, tumors were excised and fixed for immunohistochemistry, and the mice were euthanized with isoflurane overdose and cervical dislocation. Weight loss of 20% in the combination treatment group required an end of the treatment course on day 11.

### Fluorescence Microscopy

Instrumentation for fluorescence microscopy included an inverted multiphoton microscope (Bruker), a tunable titanium:sapphire laser (Coherent) for fluorescence excitation, and a Ga:AsP photomultiplier tube for collection. Time correlated single photon counting (TCSPC) electronics (SPC-150, Becker and Hickl) were used for fluorescence lifetime acquisition. NAD(P)H was imaged using an excitation wavelength of 750nm and a collection filter of 400-480nm. FAD was imaged using an excitation wavelength of 900nm and a collection filter of 500-600nm. NAD(P)H and FAD were imaged from the same fields of view. Microscopy was performed to collect images of 256x256 pixels using a 40X objective (1.3NA), 4.8 μsecond pixel dwell time, 60 second collection time, and ~10mW excitation power. Photon count rates were monitored to ensure the absence of photobleaching. A Fluoresbrite YG microsphere (Polysciences Inc.) was measured at each imaging session, and provided a lifetime of 2.10 ± 0.02 ns (n = 5), which agrees with previous reported values [26][27]. The instrument response function (IRF) was measured using second harmonic generation of urea crystals, resulting in a full width at half max of 244ps. For organoid imaging, 4–6 organoids were acquired per group. We used brightfield images from the microscope eyepiece to ensure that two-photon images accurately collect autofluorescence from the whole organoid in each field of view.

### Image Analysis

Images were analyzed using SPCImage (Becker and Hickl), as described previously [28]. Spatial binning included each pixel and the surrounding 8 pixels. The fluorescence decay curves were de-convolved with the IRF and fit to a 2-component exponential function, F(t) = α_1_e^-t/τ1^ + α _2_e^-t/τ2^. Here, α represents the contribution from each component, τ represents the fluorescence lifetime of each component, and the 2 components reflect free and protein-bound forms of NAD(P)H and FAD [26]. For NAD(P)H the short lifetime reflects the freely diffusing conformation while the long lifetime reflects the bound conformation. Conversely, for FAD the short lifetime reflects the bound conformation while the long lifetime reflects the freely diffusing conformation [11]. The mean lifetime was calculated by τ_m_ = α_1_τ_1_+ α _2_τ_2_. The optical redox ratio was calculated as the fluorescence intensity of NAD(P)H divided by the fluorescence intensity of FAD for each pixel. CellProfiler was applied to analyze images on a per-cell basis, as described previously [29]. Bar plots are consistent across 3 independent replicates.

### Heterogeneity Analysis

Heterogeneity analysis was performed as described previously [28]. Briefly, per-cell data was plotted as frequency distributions and fit to 1, 2, or 3 Gaussian curves based on the Akaike Information Criterion, where each Gaussian curve represented a cell subpopulation. Validation of this approach has shown accuracy within 10% *in vitro* [30]. The sum of the Gaussian curves was plotted. A heterogeneity index, based on a weighted Shannon diversity index, was applied to quantify cellular heterogeneity using the equation ***H*** = −∑***d***_***i***_***p***_***i***_***lnp***_***i***_ [28]. Here, *i* represents each subpopulation, d represents the distance between the median of the subpopulation and the median of all data within a group, and p represents the proportion of the subpopulation. For spatial mapping of cell subpopulations, thresholds between each subpopulation were calculated as values equidistant from the Gaussian curve means. The nucleus of each cell was color-coded according to these threshold values. Spatial heterogeneity analysis is shown for 1 replicate.

### Statistical Analysis

Bar graphs are shown as mean ± standard error. For NAD(P)H and FAD autofluorescence images, statistical significance was determined using a Student’s t-test. For tumor growth curves and immunohistochemistry statistical significance was determined using a Wilcoxon rank sum test and Bonferroni correction. Statistical tests were two-tailed, and an α of 0.05 defined statistical significance.

## Results

**Fig 1:**
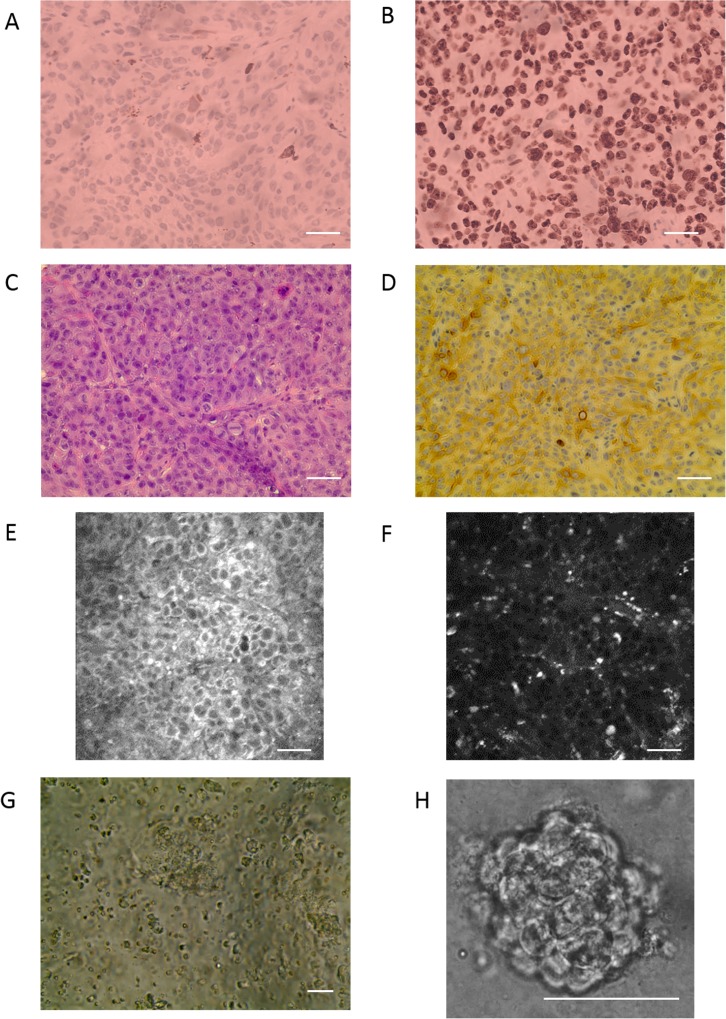
Representative immunohistochemistry, histology, and autofluorescence images of tumor tissue and brightfield microscopy for organoid generation. (A) Cleaved caspase-3 staining shows minimal apoptosis. (B) Ki-67 staining demonstrates high cell proliferation. (C) H&E staining indicates tissue composition of dense tumor cells. (D) Cytokeratin AE1/AE3 indicates positive staining of epithelial cells, which constitute the majority of the tumor. (E) NAD(P)H autofluorescence shows NAD(P)H located in the cell cytoplasm. (F) FAD autofluorescence shows punctate fluorescence signal from mitochondrial FAD. (G) Tissue was mechanically digested to create a suspension of cells. (H) Cells grow as organoids after plating the cell suspension. Scale bar = 50um.

Tumor tissue used to generate organoids was characterized with immunohistochemistry, histology, and autofluorescence imaging (Fig 1). Cleaved caspase-3 staining shows minimal cell death and ki-67 staining shows high cell proliferation (Fig 1A and 1B). H&E staining indicates tissue composition of dense tumor cells (Fig 1C). Cytokeratin AE1/AE3 staining demonstrates the epithelial status of the majority of cells (Fig 1D). Autofluorescence images show packed tumor cells with high NAD(P)H intensity localized in the cell cytoplasm and punctate FAD intensity (Fig 1E and 1F). Previous studies have confirmed that FAD intensity is localized within mitochondria [13]. These observations were confirmed in consultation with a trained pathologist. Tumor tissue was mechanically digested to break up the structural component and generate a suspension of single cells or small groups of cells (Fig 1G), which enables organoids to grow as multi-cellular aggregates (Fig 1H).

**Fig 2:**
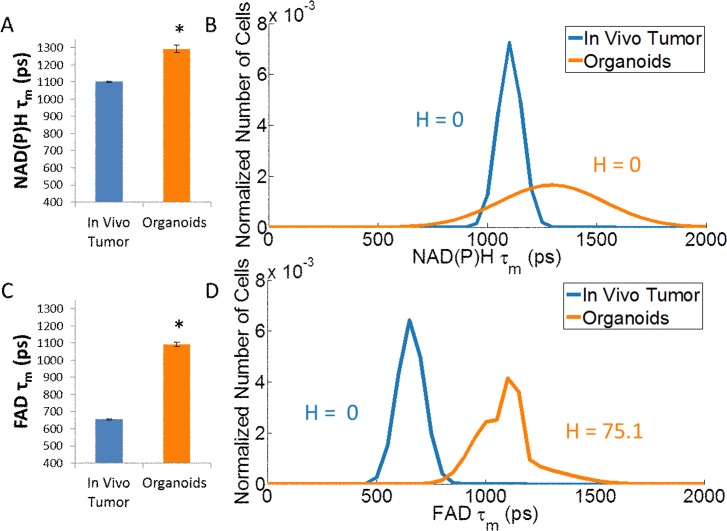
Untreated organoids and *in vivo* tumor tissue display distinct optical metabolic imaging properties. The weighted mean is calculated by τ_m_ = α_1_τ_1_ + α_2_τ_2_, where τ represents the lifetime value and α represents the contribution from each component. (A, C) Organoids exhibit higher NAD(P)H and FAD fluorescence lifetimes (τ_m_) compared with *in vivo* tumor tissue, which is explained by lower contributions of the short lifetime component (α_1_), higher values of the short fluorescence lifetime (τ_1_), and higher values of the long fluorescence lifetime (τ_2_) (See S1 Fig). (B, D) Population distribution analysis plots cellular heterogeneity for *in vivo* tumor tissue compared with organoids. The heterogeneity index (H) is similar between organoids and *in vivo* tumor based on the NAD(P)H fluorescence lifetime and increases for organoids compared to *in vivo* tumor based on the FAD fluorescence lifetime. The *in vivo* data is a subset of data published in [28]. *p<0.05, t-test, n~100–300 cells per group.

Fluorescence lifetime values are robust and self-referenced, enabling comparisons across data sets and between tumor tissue and organoids (Fig 2). Organoids exhibit higher mean lifetimes of NAD(P)H and FAD than *in vivo* tumors (p<0.05), which results from lower contributions of the short lifetime (α_1_) and higher values of the short and long fluorescence lifetimes (τ_1_ and τ_2_) (S1 Fig). Frequency distribution modeling of the fluorescence lifetimes qualitatively illustrates shifts toward higher lifetimes for organoids compared with tumor tissue. Additionally, the heterogeneity index (H) quantifies cellular heterogeneity, where an increased heterogeneity index reflects increased number of cell subpopulations, increased equality in the weights of the subpopulations, and/or increased separation between the subpopulations [28]. Organoids exhibit similar cellular heterogeneity compared with the *in vivo* tumor based on the NAD(P)H fluorescence lifetime and increased cellular heterogeneity compared with the *in vivo* tumor based on the FAD fluorescence lifetime.

**Fig 3:**
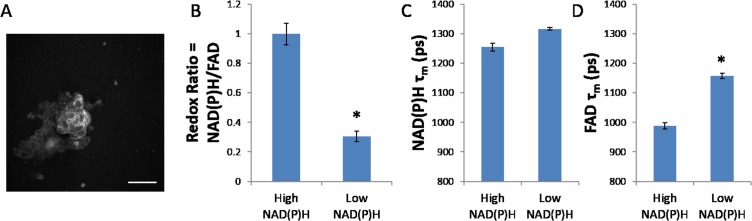
Untreated organoids contain cells with high levels of NAD(P)H intensity and cells with low levels of NAD(P)H intensity. (A) A representative fluorescence intensity image shows organoids with two levels of NAD(P)H intensity. (B) Low NAD(P)H cells exhibit a lower optical redox ratio than high NAD(P)H cells. (C) Low and High NAD(P)H cells exhibit similar NAD(P)H lifetimes. (D) Low NAD(P)H cells exhibit a higher FAD lifetime than high NAD(P)H cells. Scale bar = 50um. *p<0.05, t-test, n~50–100 cells per group.

A representative autofluorescence image demonstrates the NAD(P)H fluorescence intensity in the organoids (Fig 3). In particular, organoids exhibit populations of cells with high NAD(P)H intensity as well as cells with low NAD(P)H intensity, and these populations exhibit distinct metabolic properties. Cells with low NAD(P)H exhibit a lower redox ratio and higher FAD fluorescence lifetime, explained by a lower contribution of the short lifetime (α_1_) (S2 Fig), compared with cells with high NAD(P)H (p<0.05).

**Fig 4:**
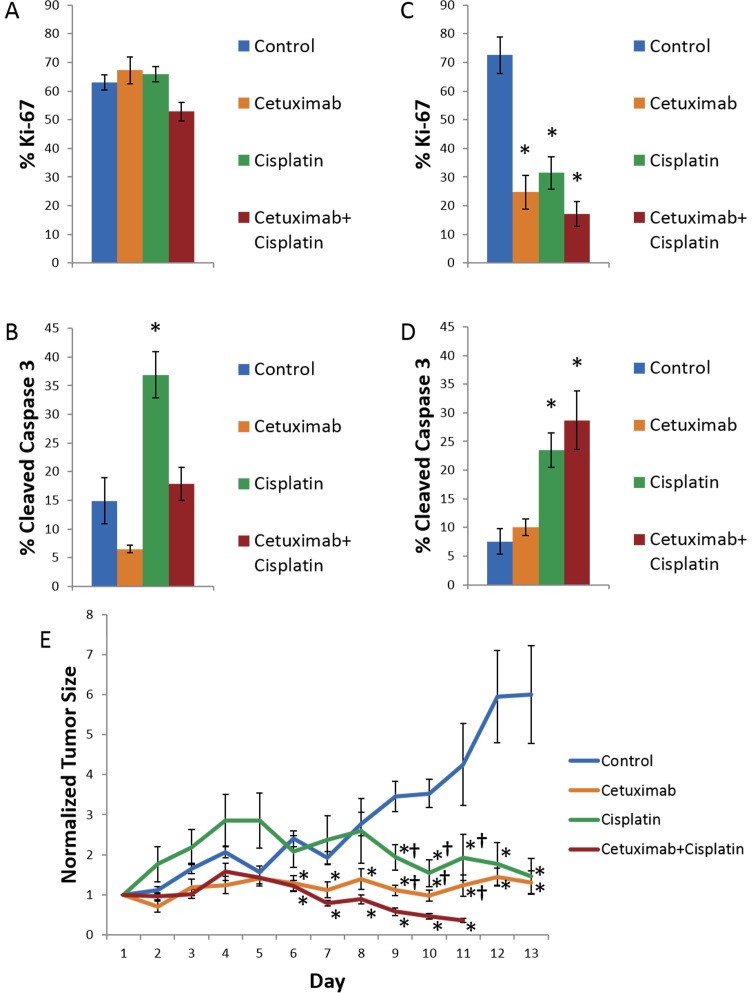
Cell proliferation and cell death were quantified using Ki-67 and Cleaved Caspase 3, respectively, in FaDu xenografts after mice were treated for 2 days or 2 weeks, and tumor growth curves show treatment effects over 2 weeks in mice with FaDu xenografts. (A) Cell proliferation is consistent across treatment groups in FaDu xenografts 2 days after treatment. (B) Cell death increases after 2 days of treatment with cisplatin. (C) Cell proliferation decreases after 2 weeks of treatment with cetuximab, cisplatin, and their combination. (D) Cell death increases after 2 weeks of treatment with cisplatin and the combination of cetuximab and cisplatin. (E) Treatment with cetuximab or cisplatin causes stable disease, whereas combination treatment causes response.*p<0.05 compared with control, rank sum test; †p<0.05, compared with combination treatment, n = 6 tumors.

Gold standard techniques validate therapeutic efficacy and measure *in vivo* response to treatment. Immunohistochemistry characterizes short-term and long-term effects of treatment on cell proliferation measured by ki-67 and cell death measured by cleaved caspase 3 (Fig 4). Two days after *in vivo* treatment, cell proliferation is consistent across all treatment groups and cell death increases with cisplatin treatment (Fig 4A and 4B). Two weeks after treatment cell proliferation decreases with cetuximab, cisplatin, and combination treatment and cell death increases with cisplatin and combination treatment (Fig 4C and 4D) (p<0.05). Tumor growth curves illustrate long-term *in vivo* response to treatment (Fig 4E). Control mice exhibit continual tumor growth, whereas mice treated with single agents of cetuximab or cisplatin exhibit stable tumor volume, and mice treated with the combination of cetuximab and cisplatin exhibit decreased tumor volume. No significant differences (p>0.1) were observed in organoid area or organoid volume between control and treated groups 24 hours post-treatment. However, significant differences in the number of cells per organoid for control vs. cisplatin groups only (p>0.1) were observed 24 hours post-treatment.

**Fig 5:**
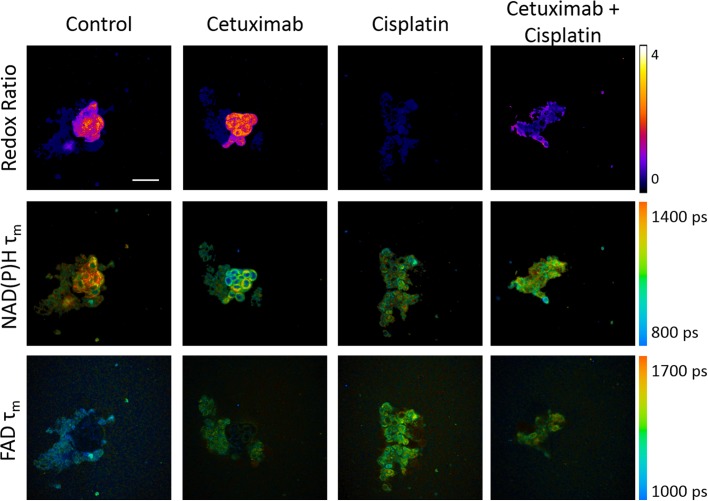
Autofluorescence images show the redox ratio and fluorescence lifetimes of NAD(P)H and FAD in head and neck cancer organoids treated for 1 day with cetuximab, cisplatin, or their combination. NAD(P)H and FAD autofluorescence images were acquired from the same fields of view, and the redox ratio (top row), NAD(P)H fluorescence lifetime (middle row), and FAD fluorescence lifetime (bottom row) were calculated. For the redox ratio and fluorescence lifetimes, blue represents a low value and yellow represents a high value (see colorbars). Scale bar = 50um.

**Fig 6:**
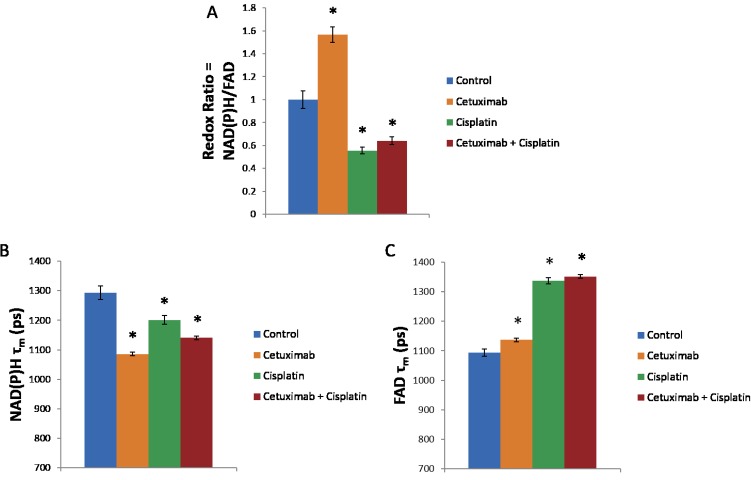
The redox ratio and fluorescence lifetimes of NAD(P)H and FAD were quantified in organoids treated for 1 day with cetuximab, cisplatin, or their combination. (A) The redox ratio increases with cetuximab treatment and decreases with cisplatin and the combination treatment. (B) The NAD(P)H lifetime decreases with cetuximab, cisplatin, and combination treatment. (C) The FAD lifetime increases with cetuximab, cisplatin, and combination treatment. *p<0.05, t-test; n~50–200 cells per group.

Representative images show organoid and cell morphology as well as relative trends in the redox ratio, NAD(P)H lifetime, and FAD lifetime for each treatment group after 24 hours of treatment (Fig 5). Optical metabolic imaging quantifies drug effects 1 day after treatment in organoids (Fig 6). The redox ratio increases with cetuximab and decreases with cisplatin and combination treatment (p<0.05). The NAD(P)H fluorescence lifetime (τ_m_) decreases with cetuximab, cisplatin, and combination treatment (p<0.05). The contribution from the short lifetime (α_1_) increases with cetuximab treatment and decreases with cisplatin and combination treatment. The values of the short and long fluorescence lifetimes (τ_1_ and τ_2_) decrease with cetuximab, cisplatin, and combination treatment (S3 Fig). The FAD fluorescence lifetime (τ_m_) increases with cetuximab, cisplatin, and combination treatment (p<0.05). The contribution from the short lifetime (α_1_) decreases with cetuximab, cisplatin, and combination treatment. The values of the short and long fluorescence lifetimes (τ_1_ and τ_2_) increase with cetuximab, cisplatin, and combination treatment (S3 Fig).

**Fig 7:**
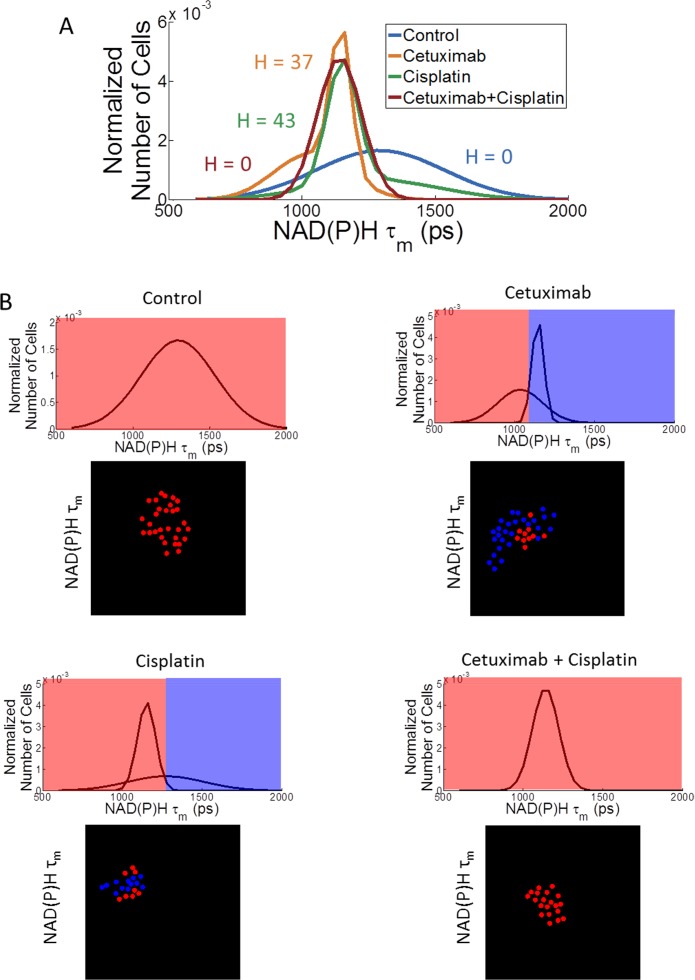
Cellular heterogeneity was analyzed based on NAD(P)H fluorescence lifetime in head and neck cancer organoids after 1 day of treatment with cetuximab, cisplatin, and their combination. (A) The sum of Gaussian curve fits provides qualitative visualization of cellular heterogeneity. A heterogeneity index (H) indicates low cellular heterogeneity for the control and combination treatment groups compared with higher heterogeneity for the single agent treatment groups. (B) Individual Gaussian curves were plotted and thresholds between the means of the Gaussian curves were color coded to inform spatial mapping, where the red and blue colors represent distinct subpopulations. The total area under the curves is equal across treatment groups. Spatial mapping provides relative locations of cell subpopulations.

Cell subpopulations describe heterogeneity within treatment groups. Heterogeneity analysis applies Gaussian fitting of per-cell data and plots the sum of the Gaussian curves, illustrating shifts toward lower NAD(P)H lifetimes after cetuximab, cisplatin, and combination treatment (Fig 7). The control and combination treatment groups exhibit one subpopulation, whereas the cetuximab and cisplatin groups exhibit two subpopulations. For each treatment group the summed area under the Gaussian curves equals one. Control and combination treated organoids display a low heterogeneity index, whereas single agent treated organoids display a higher heterogeneity index. Additionally, spatial mapping shows localization of the cell subpopulations. Each organoid contains one or both of the subpopulations, and qualitative analysis indicates that cell subpopulations are scattered throughout the organoids.

## Discussion

This study characterizes head and neck cancer organoids metabolically and in response to drugs. This approach is advantageous because it utilizes a three-dimensional model combined with sensitive metabolic measurements of treatment response. Organoids are generated from tumor tissue and grow in a three-dimensional matrix, which provides a more appropriate model than cell lines grown as monolayers on plastic [31]. Optical metabolic imaging measures early therapeutic effects and characterizes cellular heterogeneity, which is crucial for identifying resistant cells that cause resistance to treatment in patients. Overall, this technique could be adapted for high-throughput screens of treatment efficacy for anti-cancer drugs to facilitate drug discovery.

High quality primary tissue facilitates organoid growth. In particular, generating organoids immediately after tissue excision preserves tissue viability. Successful organoids grow from tissue that consists of dense tumor cells with a high proliferation rate and low apoptosis rate (Fig 1). Histological analysis indicates that the primary tissue comprises ~97% tumor characterized by proliferative, epithelial cells and ~3% stroma characterized by fibroblasts, capillaries, immune cells, and collagen. Fibroblasts exhibit distinct elongated morphology that would be apparent in culture [32]. Endothelial cells and immune cells would be expected to have short life spans and expire under organoid conditions [33]. In particular, the growth of endothelial cells is promoted by shear stress [34], which is largely absent in these cultures. This analysis suggests that the organoids comprise epithelial cells.

Protein-binding causes a conformational change in the molecular structure of NAD(P)H and FAD, which affects fluorescence quenching and the fluorescence lifetime [11]. The distinct fluorescence lifetime properties between *in vivo* tumor tissue and organoids reflect distinct protein-binding activity, including different rates that these molecules are being used in cell signaling pathways and binding to different proteins (Fig 2). These differences could result from discrete microenvironment conditions, including nutrient availability and oxygenation between *in vivo* tumors and *in vitro* cultures. This characterization highlights the utility of organoids as a complementary tool to *in vivo* imaging by enabling rapid comparisons of metabolic states between treated and control organoids generated from the same tissue.

Cell subpopulations with distinct metabolic phenotypes are present in the control organoids (Fig 3). Organoids contain cells with high levels of NAD(P)H intensity and cells with low levels of NAD(P)H intensity (p<0.05). Low-NAD(P)H cells exhibit a lower redox ratio than high-NAD(P)H cells (p<0.05), reflecting distinct metabolic characteristics. Previous studies have shown that a decrease in redox ratio corresponds to a decrease in cell proliferation [9], thus these two subpopulations of cells may have varied drug response. Additionally, these subpopulations exhibit different FAD fluorescence lifetimes and contributions from free FAD (S2 Fig, p<0.05), indicating different levels of protein-binding between these cell subpopulations. The lack of a tumor stroma in the organoids could enable these separate subpopulations of cells to grow [35].

Traditional measures of therapeutic response characterize each treatment group. Immunohistochemistry shows minimal treatment effects after 2 days of treatment, and greater treatment response after 2 weeks of treatment (Fig 4). Cetuximab has been shown to induce autophagy instead of apoptosis [21]. Overall, these results indicate that the endpoints of cell proliferation and cell death require multiple courses of treatment to resolve treatment effects. Tumor growth curves show that control tumors exhibit disease progression, the single agent treatments both exhibit stable disease, and the combination treatment exhibits treatment response (Fig 4E). These results reflect the synergistic effect of cetuximab and cisplatin, because cetuximab enhances chemotherapy-induced cell death by inhibiting DNA repair mechanisms [36]. These results agree with clinical studies of patients administered cetuximab, cisplatin, or their combination [37][38][39].

Optical metabolic imaging quantitatively demonstrates sensitivity to drug effects after 1 day of treatment (Fig 6), which is an earlier time point compared with cell death, cell proliferation, and tumor volume (Fig 4). Cetuximab treatment causes an increase in the redox ratio, which is consistent with decreased efficacy of cetuximab as a monotherapy [16][9]. Previous studies that focus on breast cancer have applied trastuzumab, an antibody therapy that targets HER2, to HER2-positive and ER-positive organoids [16]. HER2-positive organoids, which are expected to respond to trastuzumab, show a decrease in redox ratio after treatment. On the contrary, ER-positive organoids, which are expected to be resistant to trastuzumab, show no change or an increase in the redox ratio after treatment. These trends in the redox ratio for breast cancer organoids in response to antibody therapy are consistent with trends in the redox ratio in the current paper. Cisplatin treatment causes a decrease in the redox ratio, which is consistent with drug responsiveness in previous *in vitro* and *in vivo* studies [9][28]. Combination treatment causes a decrease in the redox ratio, which is previously unreported. Taken together these results suggest that a decrease in the redox ratio indicates treatment response compared with an increase or no change in redox ratio for less effective treatments.

The organoids NAD(P)H fluorescence lifetime (τ_m_) decreases with cetuximab, cisplatin, and combination treatments (Fig 6B, p<0.05), which is consistent with previous *in vivo* results [28]. The organoids FAD fluorescence lifetime (τ_m_) increases with cetuximab, cisplatin, and combination treatments (Fig 6C, p<0.05). This shows the opposite trend from previous *in vivo* results and reflects the difference in microenvironments between *in vivo* and *in vitro* conditions, including access to oxygen and nutrients, which affect the microenvironment of FAD and thus its fluorescence lifetime. Overall, these results indicate that organoids combined with optical metabolic imaging provides a unique *in vitro*, three-dimensional model that harnesses intrinsic contrast for measuring early, sensitive drug effects on a single-cell level.

Tumor heterogeneity describes multiple cell subpopulations that can respond to therapies with different sensitivities, and cells that are resistant to treatment can enable patient relapse. In particular, Gaussian fitting of cellular data can characterize cellular heterogeneity, and a heterogeneity index, H, can incorporate the number of subpopulations, evenness of subpopulations, and relative distance between subpopulations to quantify cellular heterogeneity [30][28]. Based on the heterogeneity index, organoids treated with the single agents demonstrate a higher degree of heterogeneity compared with organoids in the control and combination treatment groups (Fig 7A). As seen in the tumor growth curves (Fig 4E), combination treatment has an additive effect compared with single agent treatments and creates a uniform response in organoids based on the heterogeneity index (Fig 7A). Furthermore, spatial mapping provides insight into the relative locations of cell subpopulations, particularly for visualization of grouped versus scattered subpopulations. Representative images indicate that cell subpopulations are scattered across and within organoids. Ultimately, characterization of cellular heterogeneity could provide a powerful tool for testing drugs and drug combinations.

Head and neck cancer patients suffer from severe toxicities, serious morbidities, and mortalities, and these challenges can be addressed through improved therapies. In particular, streamlining the complex process of drug development could make a beneficial impact by efficiently identifying the most effective and least toxic drugs for development. This would reduce the time and resources spent on drugs that ultimately fail in patients and increase the success rate of clinical trials. A high-throughput drug screen based on cell metabolism and single cell analysis can address this need. Organoids provide a relevant three-dimensional model, while optical metabolic imaging provides a platform for single-cell measurements of heterogeneous therapeutic response. This study characterizes head and neck cancer organoids metabolically and measures early response to antibody therapy, chemotherapy, and combination therapy, and also identifies metabolic subpopulations of cells in the cultures. These results indicate that head and neck cancer organoids combined with optical metabolic imaging could provide a beneficial tool during drug discovery for head and neck cancer.

## Supporting Information

S1 Fig Untreated organoids and *in vivo* tumor tissue exhibit distinct NAD(P)H and FAD fluorescence lifetime components. Organoids have lower contributions of the short lifetime component (α_1_), higher values of the short fluorescence lifetime (τ_1_), and higher values of the long fluorescence lifetime (τ_2_). The *in vivo* data is a subset of data published in [28]. *p<0.05, t-test, n~100–300 cells per group.
(TIF)

S2 Fig NAD(P)H and FAD fluorescence lifetime components characterize cells in untreated organoids with low levels of NAD(P)H fluorescence compared with cells with high levels of NAD(P)H intensity. NAD(P)H fluorescence lifetime components are similar, whereas low NAD(P)H cells have lower contribution of FAD short lifetime component (α_1_).
(TIF)

S3 Fig NAD(P)H and FAD fluorescence lifetime components were quantified in organoids after 1 day of treatment. For NAD(P)H, cetuximab treatment causes an increase in the short lifetime component (α_1_), whereas cisplatin and combination treatment cause a decrease in the short lifetime component. Cetuximab, cisplatin, and combination treatments cause a decrease in the short (τ_1_) and long (τ_2_) fluorescence lifetimes. For FAD, cetuximab, cisplatin, and combination treatments cause a decrease in the contribution of the short lifetime (α_1_) and an increase in the short (τ_1_) and long (τ_2_) fluorescence lifetimes.
(TIF)
