# Supplementary figures and images for: Metabolic Imaging of Head and Neck Cancer Organoids
Source: PLoS One. 2017 Jan 18;12(1):e0170415. doi: 10.1371/journal.pone.0170415 (PMC5242529; doi:10.1371/journal.pone.0170415)

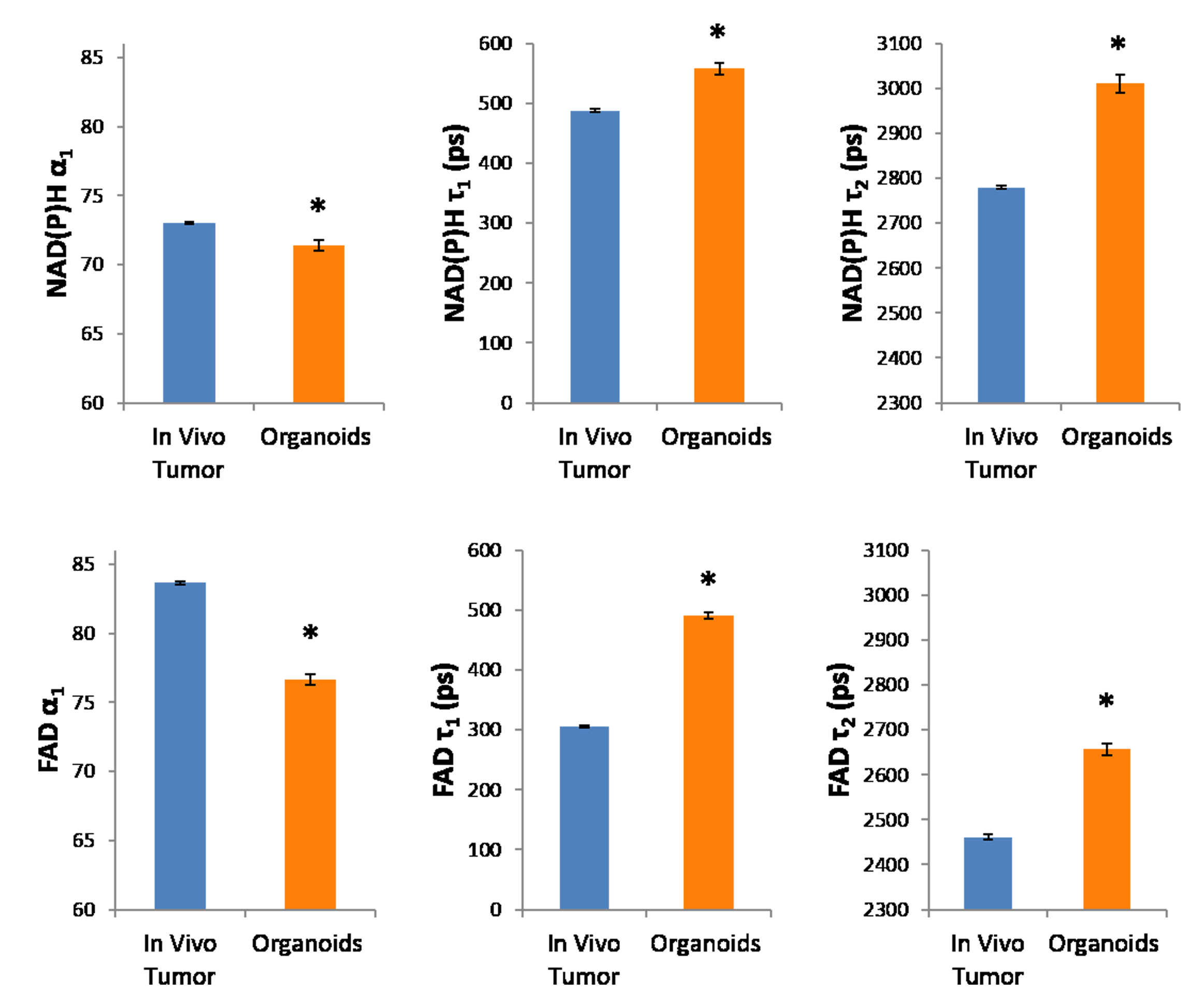

Supplement: S1 Fig — Organoids have lower contributions of the short lifetime component (α1), higher values of the short fluorescence lifetime (τ1), and higher values of the long fluorescence lifetime (τ2). The in vivo data is a subset of data published in [28]. *p<0.05, t-test, n~100–300 cells per group. (TIF) [file pone.0170415.s001.tif]

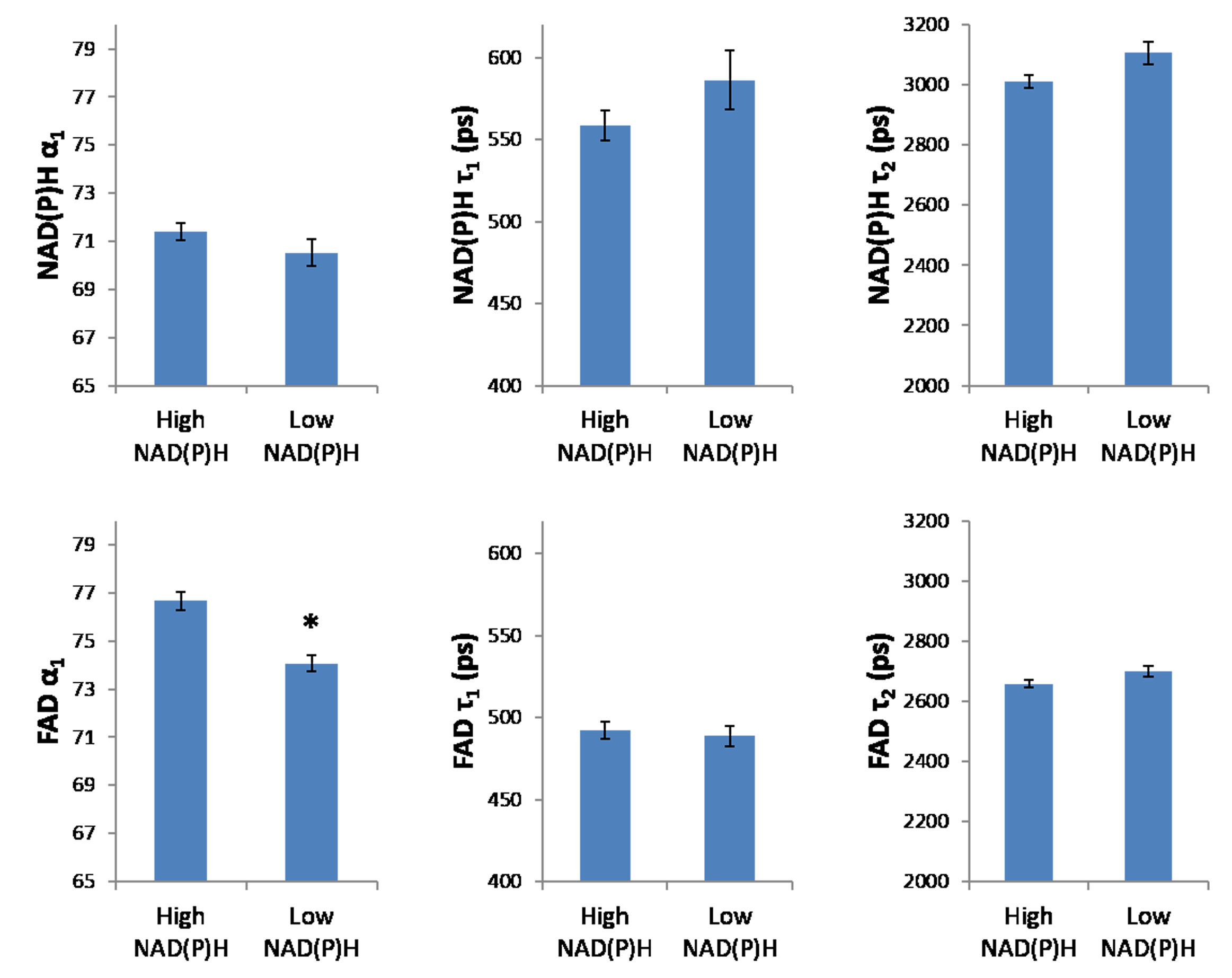

Supplement: S2 Fig — NAD(P)H fluorescence lifetime components are similar, whereas low NAD(P)H cells have lower contribution of FAD short lifetime component (α1). (TIF) [file pone.0170415.s002.tif]

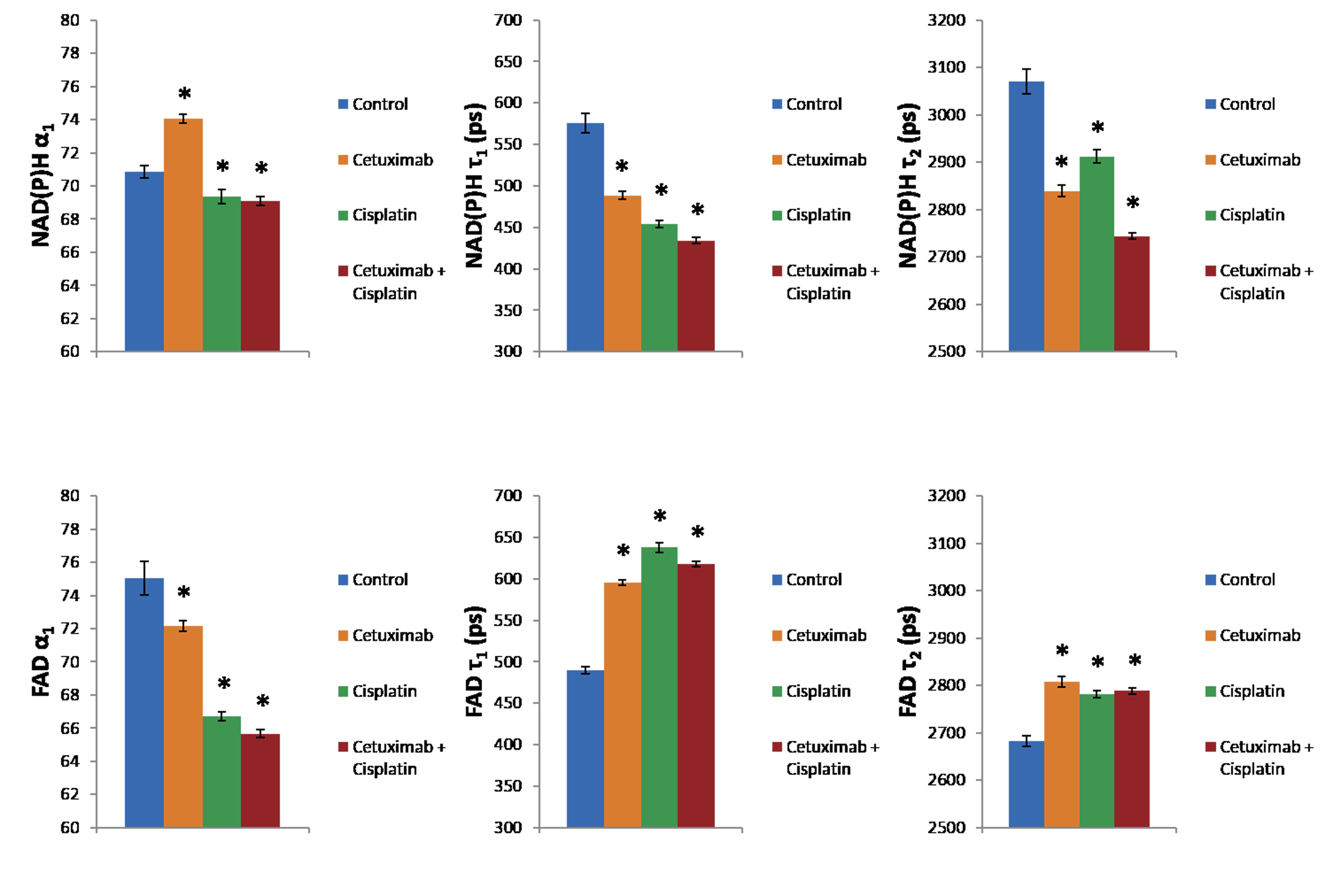

Supplement: S3 Fig — For NAD(P)H, cetuximab treatment causes an increase in the short lifetime component (α1), whereas cisplatin and combination treatment cause a decrease in the short lifetime component. Cetuximab, cisplatin, and combination treatments cause a decrease in the short (τ1) and long (τ2) fluorescence lifetimes. For FAD, cetuximab, cisplatin, and combination treatments cause a decrease in the contribution of the short lifetime (α1) and an increase in the short (τ1) and long (τ2) fluorescence lifetimes. (TIF) [file pone.0170415.s003.tif]
